# Analysis of volatile emissions from grape berries infected with *Aspergillus carbonarius* using hyphenated and portable mass spectrometry

**DOI:** 10.1038/s41598-020-78332-z

**Published:** 2020-12-03

**Authors:** Konstantinos Giannoukos, Stamatios Giannoukos, Christina Lagogianni, Dimitrios I. Tsitsigiannis, Stephen Taylor

**Affiliations:** 1 Q Technologies Ltd, 100 Childwall Road, Liverpool, L15 6UX UK; 2 Department of Chemistry and Applied Biosciences, ETH Zurich, 8093 Zurich, Switzerland; 3 Laboratory of Plant Pathology, Department of Crop Science, School of Plant Sciences, Agricultural University of Athens, 118 55 Athens, Greece; 4 Mass Spectrometry and Instrumentation Group, Department of Electrical Engineering and Electronics, University of Liverpool, Liverpool, L69 3GJ UK

**Keywords:** Analytical chemistry, Secondary metabolism, Predictive markers, Prognostic markers

## Abstract

Mycotoxins represent a serious risk for human and animal health. Οchratoxin A (OTA) is a carcinogenic mycotoxin produced by *A. carbonarius* that constitutes a severe problem for viticulture. In this study, we investigate the development of novel detection and on-line monitoring approaches for the detection of OTA in the field (i.e. out of the chemical laboratory) using advanced molecular sensing. Both stand-alone and hyphenated mass spectrometry (MS) based systems (e.g. Time-of-Flight ToF–MS and gas chromatography GC combined with MS) and compact portable membrane inlet MS (MIMS) have been employed for the first time to detect and monitor volatile emissions of grape berries infected by the fungus *Aspergillus carbonarius*. *In vacuo* (electron impact—EI) and ambient ionisation (electrospray ionisation—ESI) techniques were also examined. On-line measurements of the volatile emissions of grape berries, infected by various strains of *A. carbonarius* with different toxicity levels, were performed resulting in different olfactory chemical profiles with a common core of characteristic mass fragments, which could be eventually used for on-site detection and monitoring allowing consequent improvement in food security.

## Introduction

The impact of mycotoxins including ochratoxin A (OTA) in the vine industry has an annual cost of 1.5 billion dollars^1^, from crop loss or crop treatment^2^ to severe cases of threats to human life^3^. OTA is found amongst the various secondary metabolites from fungi such as those belonging to *Aspergillus* species. OTA has carcinogenic (Class 2B, IARC), nephrotoxic, teratogenic, immunocompromised and potentially neurotoxic attributes^4^. OTA is common worldwide and can contaminate several agricultural products such as grapes, cereals, grains of coffee, cocoa, dry fruits (*i.e.* raisins/currants), processed foods based on cereals, wine, coffee, beer as well as the juice of grapes. Provisional estimates of the Codex Alimentarius Commission, based on limited European data, suggested that red wine is the second major source of human exposure to OTA, followed by the cereals and preceding coffee and beer^5^. OTA can be produced under specific conditions of temperature and water activity^6^, from fresh grapes^7^ to raisins in their dried state^8^. The biosynthetic pathways for OTA formation have been found to be controlled from photo-chemical stimuli that regulate the expression of specific genes in terms of chemical potential imbalance^9^. More specifically, OTA synthesis from *Aspergillus carbonarius* has attracted a lot of attention, both scientifically and from a business point of view, in order to reveal its mechanisms in real vineyard conditions and to prevent OTA contamination and proliferation to other areas^10^. Measures to control OTA contamination are based on post-harvest^11^ and pre-harvest strategies. However, the management of *A. carbonarius* is very difficult, being mainly dependent on weather conditions. The last part of grape ripening is crucial for OTA synthesis. In optimal conditions for fungi, the multiple applications of systemic fungicides (*e.g.* cyprodinil/fludioxonil) are required even quite close to harvest time^12^.

The biochemical action of *A. carbonarius* in grapes was found to give secondary metabolites not only at the liquid phase (core of the fruit), but also as volatiles. So far, fifty volatile organic compounds (VOCs) were identified from *A. carbonarius* species using Gas Chromatography combined with Mass Spectrometry (GC–MS). The performance of the GC–MS was compared with and complemented by a portable sophisticated ‘electronic nose’. The two techniques were found to correlate well for predicting the various OTA levels^13^. Attempts to classify grape berries based on GC–MS have shown that *Aspergillus* species have a distinct chemical marker when compared to other fungal pathogens^14^. Using liquid chromatography (LC) coupled with ToF–MS, OTA was identified in wines from Spain^9^ and was found to be below 2 ng mL^−1^ ^15^. Volatile analysis from *A .carbonarius* has shown that various strains with moderate and high toxicity have a distinct VOC fingerprint^16^. OTA production was found to depend on temperature and water activity and especially the presence of other fungi along with *A. carbonarius*. Some fungi promote OTA production whilst others are inhibitors^17^. Modern analytical trends in volatomics for early detection of OTA, based on innovative and versatile MS solutions, could provide key solutions in detecting fungal diseases and increase food security^18^.

Portable MS and more specifically portable membrane inlet mass spectrometry (MIMS) is a powerful, simple analytical technique ideal for *in-situ* applications that offers high sensitivity (i.e. a low limits of detection—LoD), selectivity, fast and accurate analysis (within seconds), with no sample preparation requirements^19^. MIMS is based onto a three-stage sample introduction technique^20^. Sample molecules are adsorbed onto the membrane interface of the sample inlet of the MS system. They diffuse through it and then desorb into the vacuum system where they undergo thermionic ionization (if an *in-vacuo* ionization source such as an electron impact—EI is used) and mass-to-charge (*m/z*) separation^21^. Portable MS is an on-line measuring approach that can provide complete qualitative and quantitative chemical analysis of the chemical composition of any under investigation sample matrix^22^.

The aim of the current work is to investigate and report, for the first time, the time evolution of the final volatile metabolites from grape berries contaminated with *A. carbonarius* using two bench-top hyphenated and standalone MS techniques (GC–MS and ESI-ToF–MS) and one portable MS technique (MIMS). This investigation provides a more holistic approach of the temporal development of VOCs produced by the interaction of *A. carbonarius* with grape berries and allows to investigate the suitability of chemical sensing technologies for the early detection of OTA on-site. The objectives of the research include the:

1. Measurement of the VOCs emitted from grape berries contaminated with *A.carbonarius* for up to 7 days, enabling the VOCs temporal evolution profile to be determined.
2. Comparison of the VOC profile from grape berries contaminated with various strains of *A. carbonarius* (normal, non OTA producer, high OTA producer) for identification of the key volatile metabolites, enabling VOCs differentiation according to the strain.
3. Measurement of the key VOCs from the contamination of the grape berries with the normal strain of *A. carbonarius* up to 10 days using GC–MS.
4. Measurement of the OTA of the grape berries contaminated with the natural strain of *A.carbonarius* up to 10 days using ESI-ToF–MS analysis.

## Results and discussion

Analysis with the GC–MS of healthy and infected—with the strain OTA5010- grape berries, allowed the identification of the secondary produced metabolites in time (see Tables 1 and 2). A mixture of aldehydes, amides, ketones and other complex molecules showed a dynamic system of interactions between *A. carbonarius* and grape berries.

**Table 1:** Volatile compounds emitted by healthy grape berries detected with GC–MS.

Healthy grape—control				Time (days)			
Retention time (min)	Compound name	Molecular weight	Major *m/z* fragments	3	5	8	10
3.476	Glyceraldehyde	90.1	103, 147, 100, 89, 133, 117			X	X
3.658	Furfural	96.1	96, 39, 29	X	X	X	X
6.251	1,2-Cyclopentanedione	98.1	98, 69, 55, 42		X	X	X
8.551	2,4-Dihydroxy-2,5-dimethyl-3(2H)-furan-3-one	144.1	144, 101, 73, 55, 43			X	X
11.687	Aziridine-2-carbothioamide	102.2	102, 42, 30			X	X
16.122	4H-Pyran-4-one, 2,3,-dihydro-3,5-dihydroxy-6-methyl-	144.1	43, 44, 144		X	X	X
20.303	5-Hydroxymethylfurfural	126.1	97, 126, 41	X	X	X	X
30.769	Melezitose	504.4	103, 147, 361, 169, 129	X	X	X	X
37.368	Desulphosinigrin	279.3	60, 73, 43				X
60.055	9-Octadecenamide	281.5	59, 72, 55, 41		X

**Table 2:** Volatile compounds produced by infected with *A.carbonarius* -strain OTA5010- grape berries detected with GC–MS.

Infected grape				Time (days)			
Retention time (min)	Compound name	Molecular weight	Major *m/z* fragments	3	5	8	10
2.396	Butane, 1,2,3,4-diepoxy-, (- +)-	86.1	55, 29, 27		X		X
3.382	Glyceraldehyde	90.1	89, 59, 45			X	
3.524	Furfural	96.1	96, 39, 29	X	X		X
4.124	2-Furanmethanol	98.1	98, 81, 69, 53, 41		X		X
5.03	Dihydroxyacetone	90.1	31, 43, 29, 72			X	
6.317	1,2-Cyclopentanedione	98.1	98, 69, 55, 42			X	
8.535	2,4-Dihydroxy-2,5-dimethyl-3(2H)-furan-3-one	144.1	144, 101, 73, 55, 43		X	X	X
11.694	Aziridine-2-carbothioamide	102.2	102, 42, 30		X	X	X
13.381	Furyl hydroxymethyl ketone	126.1	95, 126, 39				X
16.273	4H-Pyran-4-one, 2,3-dihydroxy-6-methyl-	144.1	43, 44, 144	X	X	X	X
21.022	5-Hydroxymethylfurfural	126.1	97, 126, 41	X	X	X	X
23.066	Octanamide, N-(2-mercaptoethyl)-	203.4	57, 43, 41			X	
36.833	Desulphosinigrin	279.3	60, 73, 43			X	
55.022	Hexadecanamide	255.4	59, 72, 43	X	X		
60.115	9-Octadecenamide, (Z)-	281.5	59, 72, 55, 41	X	X	X	
63.92	Hexadecanoic acid, 2-hydroxy-1-(hydroxymethyl)ethyl ester	330.5	129, 103, 147	X	X		
66.202	Hexadecanoic acid, ethyl ester	284.5	88, 101, 70		X		
70.461	13-Docosenamide, (Z)-	337.6	59, 72, 55, 126, 41	X	X	X	

In total, we detected 72 and 93 different features of volatile metabolites emitted by the healthy and the infected grape berries for three biological replicates respectively, with 10 common volatile metabolites characteristic of the healthy grapes and 18 VOCs characteristic of the infected. The healthy grape berries were characterized by 10 isolated compounds (see Table 1) and the contaminated berries by 18 compounds (see Table 2). Both cases of healthy and infected grape berries shared 9 common metabolites. The infected berries showed the existence of 9 exclusive compounds (not found at the healthy berries) and the healthy berries demonstrated the existence of one exclusive compound (melezitose, see Table 1). Melezitose, a trisaccharide composed of fructose and glucose was found to be produced only at the healthy berries from the start of the experiment and to persist up to the 8th day. Melezitose was not found at the contaminated grapes. Based on the^23^, melezitose can be hydrolysed by enzymes isolated from *Aspergillus* species such as *Aspergillus niger .* These enzymes are capable of hydrolyzing honeydew sugars of polymers containing glucose and fructose, preferably trehalulose and melezitose. We can infer that melezitose: (1) was produced from infected grapes before day 1 and/or (2) was degraded by *A. carbonarius* during fungal colonisation. Furfural, 5-hydroxymethylfurfural, 4H-pyran-4-one and 2,4-dihydroxy-2,5-dimethyl-3(2H)-furan-3-one were found to be present at both healthy and contaminated grape berries for the entire duration of the infection experiments (see Tables 1 and 2). Other compounds were also common at the healthy and infected berries but only detected one or 2 days of sampling. Furfural and 5-hydroxymethylfurfural were found to be present whether the grape berries were infected or healthy. This observation shows that specific metabolic routes of the aging process of the grapes remain stable even during the infection. On the other hand, compounds like the ones with retention times 63.92, 66.202 and 70.461 min for the infected berries provided evidence for the interactions of *A. carbonarius* with the berries’ substrate. These interactions led to more complex compounds with higher molecular weight but similar backbone. Compounds with retention times 63.92 and 66.202 min had as backbone the hexadecanoic acid and compounds with retention times 55.022 and 70.461 min had as backbone a similar saturated long chain structure to the hexadecanoic acid. The plant cutin monomer 16-hydroxy hexadecanoic acid can be activated by sensor proteins in fungi, that activates a MAPK-dependent signaling pathway leading to a genome wide transcriptional reprograming in the fungus in order to help its invasion and colonisation in the plant tissue^24^. The differential interactions of *A. carbonarius* with the berries appeared to start from the 1st day of the infection. These interactions produced three exclusive compounds (hexadecanamide, hexadecanoic acid, 2-hydroxy-1-(hydroxymethyl)ethyl ester and 13-docosenamide, (Z)-) that were not present at any period at the healthy grapes. 13-docosenamide, (Z)- was found to be present until the 8th day of the infection. During the rest of the experiment, specific compounds were found to characterise the infected berries, in contrast to the healthy grapes. During the 5th, 8th and 10th day, in the case of infected grapes five (butane, 1,2,3,4-diepoxy-, (- +)-; 2-furanmethanol; hexadecanamide; hexadecanoic acid, ethyl ester; 13-docosenamide, (Z)-), three (dihydroxyacetone; octanamide, N-(2-mercaptoethyl)-; 13-docosenamide, (Z)-) and three (butane, 1,2,3,4-diepoxy-, (- +)-;2-furanmethanol; furyl hydroxymethyl ketone) compounds were found, correspondingly, to differentiate them from the healthy grape berries. Most of the long chain compounds were found to be absent at the 10th day of the contamination and ring based compounds were formed. Hexadecanamide and 2-Hexadecenamide are members of the poorly known family called fatty amides that are are implicated in early seedling development and in plant–microbe interactions^25,26^. The different compounds could act as a fingerprint of the contamination per day and the differentiating lines of the infected from the healthy grapes. In contrast to the healthy berries, the infected berries were found to produce 3 distinct amides, 2 esters, 2 ketones, 1 furan and a diepoxy compound. It is noted that esters were produced up to the 5th day of the contamination, while at 8th and 10th day the esters are absent and ketones are present. Representative ion chromatograms are shown in the supporting information (Figure S1).

Butane, 1,2,3,4-diepoxy-, (- +)-, or simply 1,2:3,4-diepoxybutane (DEB), was found in our study (see Table 2) as a liquid product, possibly, from the interaction of *A. carbonarius* with the grape berries. A biosynthetic precursor of DEB might be a diepoxy form that was found from the metabolomic analysis of *A. melleus*^27^. Another possible source might be the process of fumigation of grapes using hexachloro-1,3-butadiene^28^, that remained in the grapes and created the basis for DEB formation. The exact mechanism of DEB formation for the case of grapes contaminated with *A. carbonarius* is unknown^29^. DEB was found to be weakly mutagenic in rats^30^, and to induce genetic alterations at any stereoisomer form in humans^31^. 2-Furanmethanol was found to be formed naturally from fungi like *Oxyporus latem arginatus*, and to inhibit the growth of mycelium in microorganisms such as *Alternaria alternata, Botrytis cinerea, Fusarium oxysporum f.sp. lycopersici* and *Colletotrichum gloeosporioides*^32^*.* 2-Furanmethanol was found at the final stages of Merlot wine production in Brazil^33^. It is accepted by WHO (World Health Organisation) as a flavouring additive, with the daily uptake not to exceed 53 mg/kg^34^. However, 2-furanmethanol was found to induce cancer to the nasal system and the kidneys according to USA reports^35^, while according to WHO can cause urinal problems in rodents^36^. 2-Furanmethanol, or as commonly stated furfuryl alcohol was found to be a product from the hydrolysis of acetate and to be stable in wines^37^.

Furfural was reported to increase with ripeness in Merlot wines, that along with furfuryl alcohol was stated not to be a health hazard^38^. It was mostly present in wines subjected to smoke^39^. However, furfural, similarly to the furfuryl alcohol, has an impact in the olfactory system of rats^40^ and causes lung infections in humans^41^. The bioaccumulation of furfural in other studies was found to be even lethal, or at least be the cause for the genetic degradation and death in rats^42^. Despite the fact that furfural is a natural product from the dehydration of xylose^43^, in fact induces irritations to the human nasal system or even hepatitis due to long term exposure^44^. Recently, the International Agency for Research on Cancer (IARC) has identified both furfural and 2-furanmethanol to be in the group 3 of the classsification (as potent cancer inducing substances), due to the lack of adequate data to support the carcinogenic activity^45^. Dihydroxyacetone was found as a natural product from fungi like *B. cinerea* during the metabolization of glucose^46^. Dihydroxyacetone has been found to be a sign of must infection (at high oxygen concentrations and alcohol < 5%v/v) and not of alcoholic fermentation^47^. In addition, high concentrations of dihydroxyacetone could lead to increased sulfites and to change the sensory properties of the wine^47^. Dihydroxyacetone was found to be a part of the OTA production of *Penicillium citrinum* and to be controlled by the pH of the growing medium^48^. In our study, the production of dihydroxyacetone (amongst other chemicals) simultaneously with the OTA production is presented for the first time for the case of *A. carbonarius*. The correlation of OTA with the characterisation of the associated produced metabolites from the fungi was stated as key for the clear hazard analysis due to mycotoxins^49^. The simple handling of fungi like Aspergillus could be lethal if precaution measures do not monitor the OTA production at any stage of Aspergillus growth^49^.

Furyl hydroxymethyl ketone or hydroxymethylfurfural (HMF), along with OTA, has been detected at a previous study in sun-dried grapes contaminated with *Aspergillus spp*. due to non-enzymatic, condensation reactions between the sugars and the amino-acids^50^. The same study has stated that HMF in fresh foods is close to zero, which is not in line to our findings during contamination of fresh grapes with *A. carbonarius*. HMF has been found to be an indicator of food degradation to heat^50^, and to induce genotoxicity, mutagenicity but also to have beneficial impact to the human health (*e.g*. antiallergic and antioxidative)^51^. Despite the last positive feedback for HMF, its genotoxicity and carcinogenicity has placed HMF under further studies primarily *in-vivo*^52,53^. 4H-Pyran-4-one, 2,3-dihydroxy-6-methyl- or 2,3-dihydro-3,5-dihydroxy-6-methyl-(4H)-pyran-4-one (DDMP) was found in our study and has already been found as a bioactive compound from *A. niger* using GC-MS^54^. DDMP in fact is a volatile during the process of wine fermentation^55^, but there is also evidence for its mutagenicity and impact at the DNA^56^.

Hexadecanoic acid, 2-hydroxy-1-(hydroxymethyl)ethyl ester, that we found has also been isolated from medicinal plants as bioactive compounds^57^ and from *Aspergillus spp*.^58^. Hexadecanoic acid, ethyl ester or ethyl hexadecanoate (EH) has been isolated from grapes infected with *A. carbonarius*^16^. It is well established in plants, that fatty acid (FA) metabolic pathways play significant roles in pathogen defense. Both 16- and 18-carbon FAs participate in plant defense to modulate basal, effector-triggered, and systemic immunity in plants^59^. EH has also been isolated from *A. niger* as a novel metabolite when grown *in vitro*^60^. Studies have revealed the correlation of OTA producing strains like *A. alliaceus* with the EH that was detected as volatile^61^. EH was amongst the dominant esters in contaminated grapes when infected by powdery mildew and was found to persist in the produced wine over other esters^62^.

13-Docosenamide, (Z)- has been isolated only from toxigenic fungi like *A. flavus* and was stated to differentiate from the non-toxigenic fungi^63^. However, 13-Docosenamide, (Z)- appeared to have no hazards in rats or humans^64^, with the potential to act as antidepressant and andanxiolytic substance^65^.

**Figure 1:**
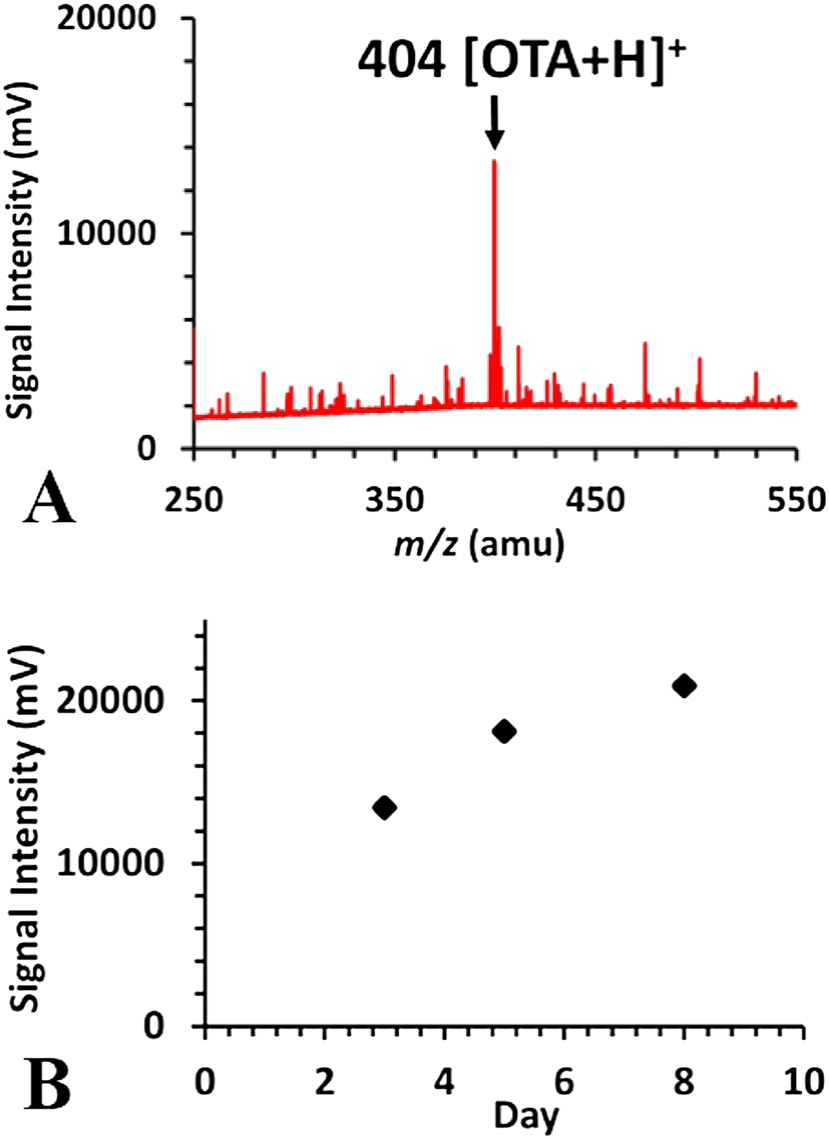
(**a**) Representative ESI-ToF mass spectrum (in the positive ion production and detection mode) of a diseased grape producing OTA in day 3 after the infection and (**b**) time evolution of the signal intensity of the m/z 404 during a period of 8 days after the infection.

A high-resolution MS system equipped with an ESI source, was utilised to detect in positive and negative ion modes the presence or absence of OTA in grapes infected by *A. carbonarius*. ESI was selected due to its soft ionization scheme that allows a clear molecular identification of compounds of interest (*e.g.* OTA that has a low vapour pressure). In addition, the TOF mass analyser has a wider mass range (*m/z* 1–1000 amu) compared to the currently available detection *m/z* range of the mass analyser of our portable MS system. The direct introduction of the generated ions into the ion guides and the mass analyser allows a smooth ion transmission for characterization. Figure 1A shows a representative mass spectrum in the positive mode of OTA present in a grape infected by *A. carbonarius* in day 3 after the appearance of conidia. The signal intensity from the ToF–MS represents the concentration of the protonated [OTA + H]^+^ and is plotted against the mass *m/z* value. Figure 1B shows how the signal of the characteristic ion of OTA evolves during a time period of 8 days. We observe a signal increase of 36% from day 3 (1st measurement) until day 8 (last measurement).

**Figure 2:**
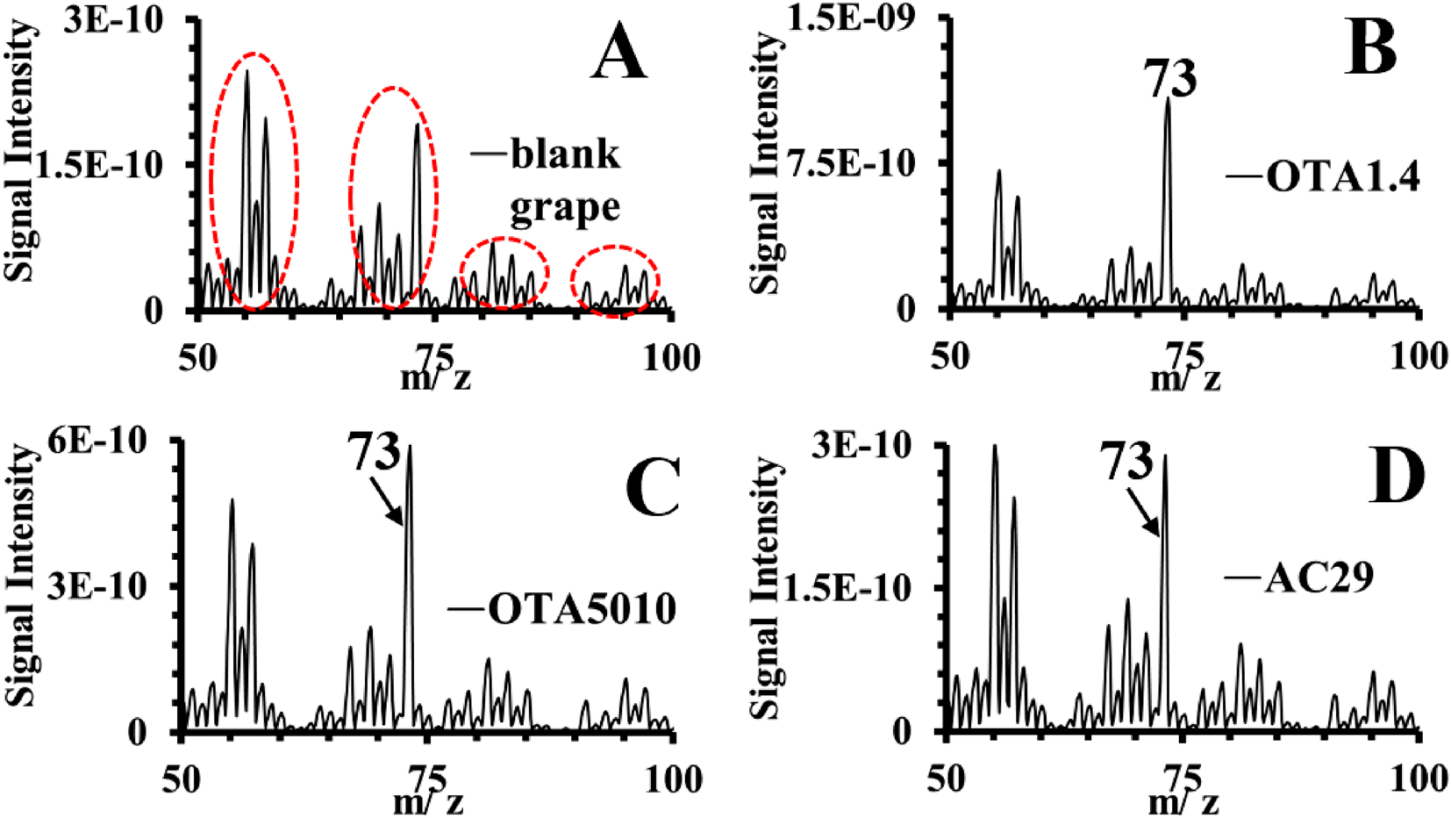
Representative mass spectra of the various strains of *A. carbonarius* at the first day of the infection experiments.

**Figure 3:**
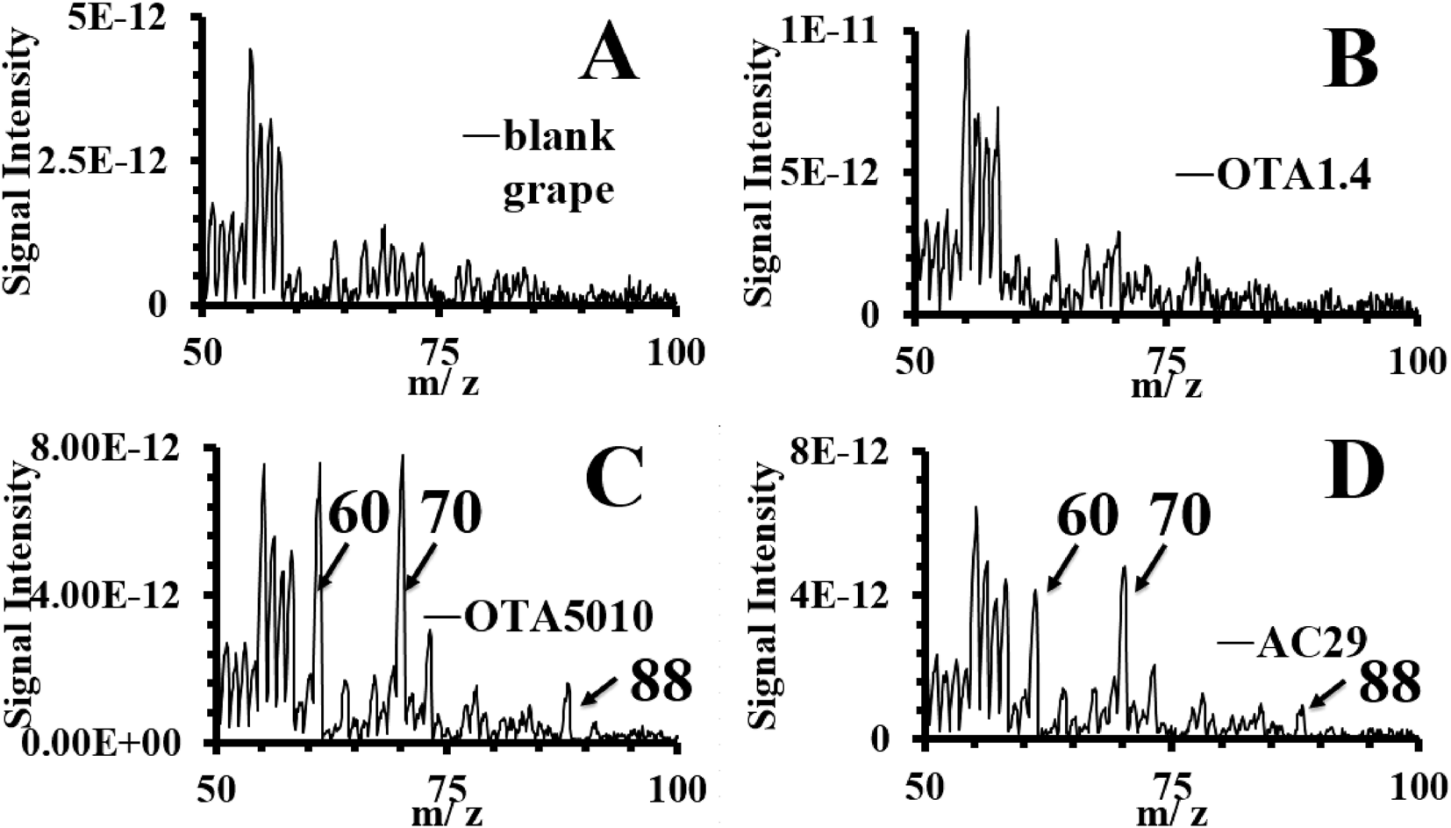
Characteristic mass spectra of the various strains of *A. carbonarius* 7 days after the contamination experiments.

This section describes the results from the VOCs detected using a portable membrane inlet mass spectrometer. Figures 2 and 3 give two snapshots of the VOCs profile at the 1st day after the infection and after 7 days respectively. It must be highlighted that the detected VOCs consist of the gaseous metabolites during the growth of *A.carbonarius* having as substrate the grape berries and thus they are the result of their interaction.

**Figure 4:**
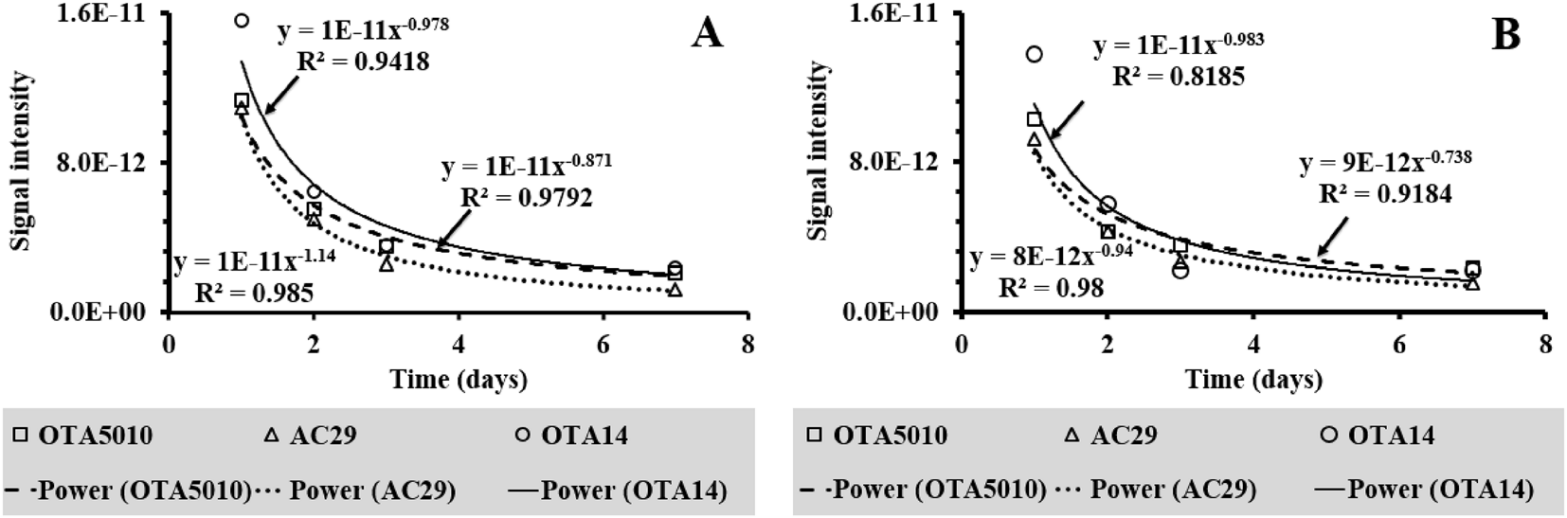
Time evolution of peaks 69 (**A**) and 73 (**B**) from the MIMS spectra for the investigated strains of *A.carbonarius.*

In generally, the graphs of the VOCs profiles appear to decline from 1 to 7 days. The decline was found to follow similar trends in time for all the *A.carbonarius* strains as shown characteristically for peaks at *m/z* 69 amu and at *m/z* 73 amu (see Fig. 4). Those peaks are used here only indicatively to show the time evolution of the signal intensity and are not characteristic of a specific strain. As shown in previous studies where MIMS has been applied^66^, the overall decline of the intensity of the graphs of the mass spectra can be correlated with the linear decline of the concentration of the volatile metabolites released from the interactions of the grape berries with *A.carbonarius*. The decline according to Fig. 4, seemed to reach a plateau after 7 days, showing that the metabolic activity of all strains has reached a semi-steady state, which would be preserved until the berries substrate has been consumed.

**Figure 5:**
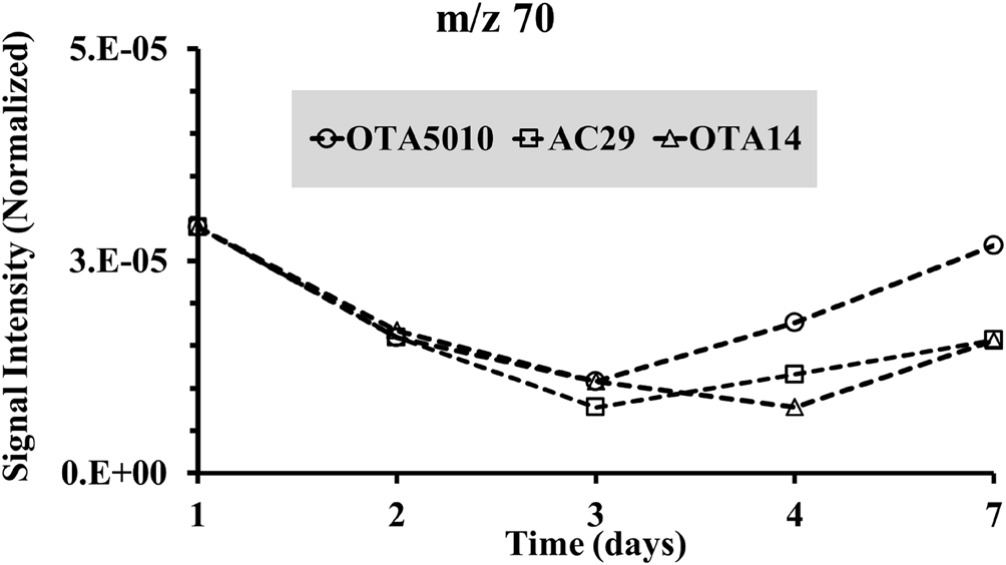
Time evolution of peak m/z = 70 amu from the mass spectra from MIMS for the strains of *A.carbonarius* (the normalization was with reference to the signal intensity of nitrogen with peak at *m/z* 28 amu).

**Figure 6:**
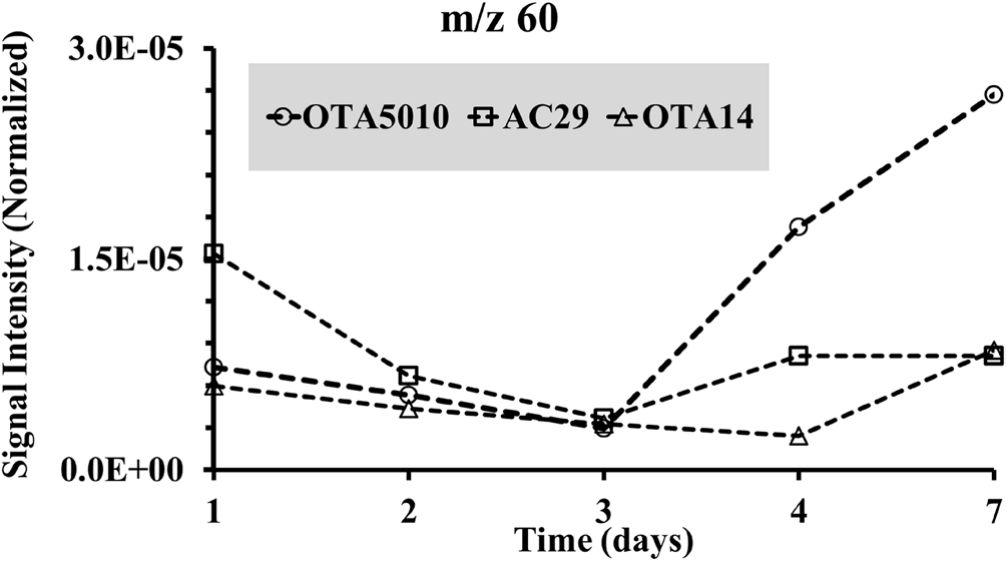
Time evolution of peak m/z = 60 amu from the mass spectra from MIMS for the strains of *A.carbonarius*(the normalization was done with reference to the signal intensity of nitrogen with peak at *m/z* 28 amu).

At the start of the infection a significant number of mass fragments appeared for all the *A. carbonarius* strains in comparison to the uninfected grapes (see Fig. 2A). A peak at *m/z* 73 amu was observed for the non-producing OTA strain, with the intensity to be three fold greater in comparison to the OTA producing strains. The three peaks at 55, 56 and 57 amu of the uninfected grapes appeared to be the same at the infected grapes, being threefold greater for the non-producing OTA strain and twofold greater for the naturally isolated strain. As observed from Fig. 2, the mass spectra appear to form specific groups of peaks (such as the peaks at *m/z* 55, 56 and 57 amu), with the groups to be separated clearly the one from the other. An example of such groups is shown in Fig. 2A for the healthy grape berries that also exist at the infected berries. The presence of such metabolites in groups occurred until the 3rd day of measurements when OTA started to be produced (see Fig. 1A). The time evolution of the volatiles after 3 days of infection, exhibited a decrease similar to the trends shown in Fig. 4. When OTA was produced at the 3rd day of the infection, the specific groups of peaks shown in Fig. 2, were replaced by non-symmetrical spectra (see Fig. 3). Evidence from specific peaks from MIMS measurements has shown a minimum, as far as the relative signal intensity is concerned, at the 3rd day of the infection and then an increase of the intensity (see Figs. 5 and 6). That minimum was observed primarily for strain OTA5010 showing for the first time, evidence between specific peaks captured using MIMS with OTA detection from ESI-ToF–MS.

Strain AC29 exhibited similar peaks with strain OTA5010 for each duration of the infection experiments. At the start of the infection, the strain that was not producing OTA, showed the most intense peaks in comparison to the other two strains by an order of magnitude (see Fig. 2). The lack of intense and distinct peaks for strain AC29, could be assigned to the adaptation stage for the production of OTA. These peaks which were more intense for the non- OTA producing strain, could correspond to metabolites that strain AC29 has used as biosynthetical precursors towards OTA production.

After 7 days, all strains showed distinct peaks at 47 amu and 49 amu in contrast to the blank grape berries. It is possible that such correspond to fragments from the compounds detected exclusively at the infected grape berries using GC–MS (see Table 2). The OTA producing strains (OTA5010 and AC29) showed characteristic peaks at *m/z* 60 amu, *m/z* 70 amu and *m/z* 88 amu (see Fig. 3C,D). The three peaks of the OTA producing strains, that were found to increase their intensity from the day 3 of the contamination onwards, are reported for the first time as an *in-situ* fingerprint of the OTA production in infected grape berries with *A.carbonarius*. The time evolution of peaks at *m/z* 60 amu and *m/z* 70 amu is shown in Figs. 5, 6, respectively. The peak at *m/z* 88 amu is not shown since it was detected at the 7th day only for the OTA producing strains. Overall from the onset of the infection to the 3rd day of our experiments, when OTA was detected, there was a 99.7% decrease of the signal intensity (see Fig. 4). Here we chose not the half life (50% decrease) of the signal intensity of the graphs from Fig. 4 (that corresponds to the 2nd day of the infection), but the critical day of OTA production. That observation is made on the basis that measurements with MIMS were regular (*i.e.* taken on a daily basis) and that the overall mass spectra decline in time. In case that an $$i $$ measurement gives at least three peaks with increased signal intensity than the $$i - 1$$ measurement, then we showed that OTA has already contaminated the grape berries. Hence, we conclude that preventive measures, should target just after the 2nd day of the infection in order to avoid OTA production at least at the levels that we detected using the ToF–MS.

## Conclusions

Herewith, we report preliminary findings on the detection of the OTA and VOCs associated with its presence on grape berries during 10 days after the infection with *A. carbonarius* using three complementary MS techniques. We utilized benchtop GC–MS (with an EI ion source) and high-resolution ESI-ToF–MS and a compact portable MIMS system.

1. GC–MS provided an olfactory chemical profile of the diseased grapes providing a clear molecular identification of the detected compounds. Overall compounds with retention times greater than 23 min appeared to transform to compounds with retention times less than 23 under the analytical conditions of our study.
2. Three to five compounds are distinct per day for the infected grape berries with the *A. carbonarius* when compared to the healthy berries. Those exclusive compounds detected using GC–MS could give rise to the three characteristic peaks observed with MIMS after OTA production at the 3rd day of infection.
3. ESI-ToF–MS gave very promising results for early detection of OTA and its progression using a soft ionization approach that allows the ionization of OTA and allows its identification as protonated or deprotonated compound.
4. Our results show that the initial two days after the infection with the *A.carbonarius* are critical for applying preventive measures before before OTA is detected. On the third day, when OTA is produced, bioaccumulation of OTA at the grapes could pose a health hazard, with economic implications also.
5. Mass spectra measured with MIMS correlated well with the time for OTA detection using ESI-ToF–MS. Simultaneously, MIMS showed evidence of the compounds detected using GC–MS. The combination of all three techniques showed which compounds testify to the presence of OTA. Field studies of their biochemical pathways should allow effective inhibition of OTA synthesis.

The data obtained using our portable MIMS equipped with an electron impact (EI) ionization source gave characteristic peaks to be further investigated using chemometric algorithms to create a mathematical model that will link target mass fragments with the identification of OTA during in-field operations. The results demonstrate the possibility of using MS to detect plant diseases caused by ochratoxigenic fungi and OTA presence in grapes *in-situ*.

Our future plans is to apply the developed portable MIMS system in field activities and measurements to detect and monitor volatile compounds emitted by diseased grapes in real-conditions. We also plan to modify our portable MS system in such a way as to bypass the EI source and couple it with an online miniature ESI source that will allow a clear real-time identification and characterization of the OTA on-site. This will simplify the rapid detection and monitoring of OTA on-site and will benefit end-users.

## Experimental

### Materials, methods and infection assays

Fresh Greek grapes of Crimson Seedless variety were bought from a commercial UK supermarket and selected for the contamination experiments on the basis of no scratches and to be similar in size. Three strains of *A. carbonarius* were used for the contamination experiments: (1) OTA5010 was the reference strain, (2) AC29 is a high OTA producing strain^67^ and (3) OTA1.4 is a genetically modified strain (*ΔAclaeA1.4*) that is not an OTA producer because of deletion of the *AclaeA* gene^68^. Two non-infected grape samples (with and without immersion in water) were used as reference negative controls. For each case three petri dishes were used with 6 grapes per sterile dish for assessing the reproducibility of the results. OTA standard of 40 ppb was purchased with the AgraQuant Ochratoxin ELISA test by Romer Labs Inc. (Getzersdorf, Austria). In order to compare the effect of the three above mentioned *A. carbonarius* srains in terms of volatile profile, the grapes were sterilized with 10% NaClO for 10 min, washed with sterile distilled water, placed in 70% ETOH for three min and washed with ddH_2_O. The inoculum of 10 μl of 10^6^ conidia ml^−1^ for each tested strain was placed in an artifial 2–3 mm wound on the surface each grape.

### Characterisation methods

Three complementary benchtop and portable MS approaches (hyphenated and standalone) equipped with *in-vacuo* and ambient environment ionization schemes (EI and ESI) and two different mass analysers (quadrupole and time-of-flight) were used to investigate the presence or absence of OTA in grape berries as well as to detect and investigate the chemical profile and its evolution of the volatile emissions produced by grapes infected by various strains of *A. carbonarius*.

#### GC–MS

For the detection of the VOCs emitted from the: (a) healthy and (b) infected by the fungus *A. carbonarius (OTA-5010)* grapes, an Agilent gas chromatography–mass spectrometry system (GC–MS—7890B/5977A) was used. The purpose was to study the volatile organic compounds emitted on the 3rd, 5th, 8th and 10th day after the infection with the fungal spores (conidia).

The samples were introduced to GC–MS by micro syringes in the liquid form. For the experiments discussed in this section, control and infected grapes were transferred into 15 mL vials and mixed with high purity methanol (99.999%). The samples were then blended until fine and filtered. Liquid methanolic solutions (1.5 μL) were then introduced into the GC sample injection port for further analysis. The column that was used was a HP-5MS UI purchased from Agilent. Its geometrical characteristics are: 30 m × 0.250 mm × 0.25 μm, with temperature limits from − 60 °C to 350 °C. GC separation involved a ramped increase of the temperature of the GC oven from 50 °C up to 350 °C with step temperature rate of 3 °C/min and flow rate of 5 mL/min. The sample inlet at the GC and the sample transfer line from the GC to the MS was heated at 250 °C, the EI ion source at 230 °C, while the quadrupole mass analyzer was constantly kept at 150 °C. The Agilent 5977A Mass Spectrometer was equipped with an extractor ionisation source, a heated gold quadrupole and triple-axis detector for enhanced (ultratrace level: low fg to low ng) sensitivity, clearer spectral appearance and compound identification. The mass range was from 1 to 1050 amu with a better than 0.1 amu/48 h mass axis stability. The GC–MS was tuned using a novel one-click autotuning system for PFTBA. Data acquisition, interpretation and analysis was carried out using the Mass Hunter and Chem Station software^69^. The Wiley 10th and NIST libraries were utilised for the chemical characterization and identification of the chemical compounds of the samples under examination. The repeatability of the measurements had less than 1% relative standard deviation (RSD).

#### High-resolution ESI-ToF- MS

A Waters Xevo G2 QToF-MS equipped with an electrospray ionization source (ESI), was used to study the presence and progression of the OTA in a) control and b) infected grapes. For these experiments, we utilized both the positive and the negative ion production and detection mode. The working solutions were prepared in methanol and in total 4 measurements were obtained on the 3rd, 5th, 8th and 10th day after the infection with *A. carbonarius* conidia. The positive mode of the ESI source was set at + 2.5 kV, whereas the negative mode at − 2.7 kV, and the ESI capillary had an ID of 50 μm.

#### Portable MIMS

A portable, membrane inlet mass spectrometer (MIMS) was used for the on-line detection and monitoring of volatile organic compounds (VOCs) from the contaminated grapes. MIMS offers high sensitivity (to ppt levels)^70^, fast and accurate analysis (within seconds)^22^, with minimum to no sample preparation. The MIMS sample inlet operates using the principle of pervaporation separation through polymer membranes which has been shown to be particularly useful for VOC measurement^71^.

The gaseous metabolites were collected via a sampling probe consisted of a thin polydimethylsiloxane (PDMS) sheet^66^. The sampling method consisted of the placement of the sampling tube in the headspace area above the petri dishes hosting the grape berrie with no treatment of the grapes in order to simulate the field sampling. It is highlighted that the sampling probe did not touch any of the berries and recorded continuously the emitted VOCs in the gas area above the grapes in near-real time. The technique is therefore non-destructive and non-contaminative. Physically, the grape volatile molecules are being adsorbed onto the surface of the membrane and then after permeation, desorbed into the MS chamber. The PDMS membrane allowed for the fast diffusion of non-polar VOCs from the biochemical activity of *A.carbonarius* and the near-real time detection using a triple filter quadrupole mass analyser (QMS)^72^. Each full MIMS scan (*m/z* 1–200 amu) takes approximately 3 min which effectively monitors the VOC profile in near real time. Details of the MIMS provision are given in a previous study^66^. The main parts of the MIMS system used during our experimental investigation have been described in detail previously^19–21,71,73^.

## Supplementary information

Supplementary Information
