# Supplementary material for: Analysis of volatile emissions from grape berries infected with Aspergillus carbonarius using hyphenated and portable mass spectrometry
Source: Sci Rep. 2020 Dec 3;10:21179. doi: 10.1038/s41598-020-78332-z (PMC7713432; doi:10.1038/s41598-020-78332-z)
Supplement: Supplementary file 1 — Supplementary Information [file 41598_2020_78332_MOESM1_ESM.docx]

**Supporting Information**

**Analysis of volatile emissions from grape berries infected with *Aspergillus carbonarius* using hyphenated and portable mass spectrometry**

Konstantinos Giannoukos^a^, Stamatios Giannoukos^b,^*, Christina Lagogianni^c^, Dimitrios I. Tsitsigiannis^c^ and Stephen Taylor^a,d,^*

^a^ Q Technologies Ltd, 100 Childwall Road, Liverpool, L15 6UX, United Kingdom

^b^ ETH Zurich, Department of Chemistry and Applied Biosciences, 8093 Zurich, Switzerland

^c^ Agricultural University of Athens, School of Plant Sciences, Department of Crop Science, Laboratory of Plant Pathology, 118 55, Athens, Greece

^d^ Mass Spectrometry and Instrumentation Group, Department of Electrical Engineering and Electronics, University of Liverpool, Liverpool L69 3GJ, United Kingdom

*Corresponding authors: e-mail: stamatios.giannoukos@org.chem.ethz.ch & S.Taylor@liv.ac.uk.

Keywords: Mass Spectrometry, *Aspergillus carbonarius*, Ochratoxin-A, Volatilomics, Mycotoxins


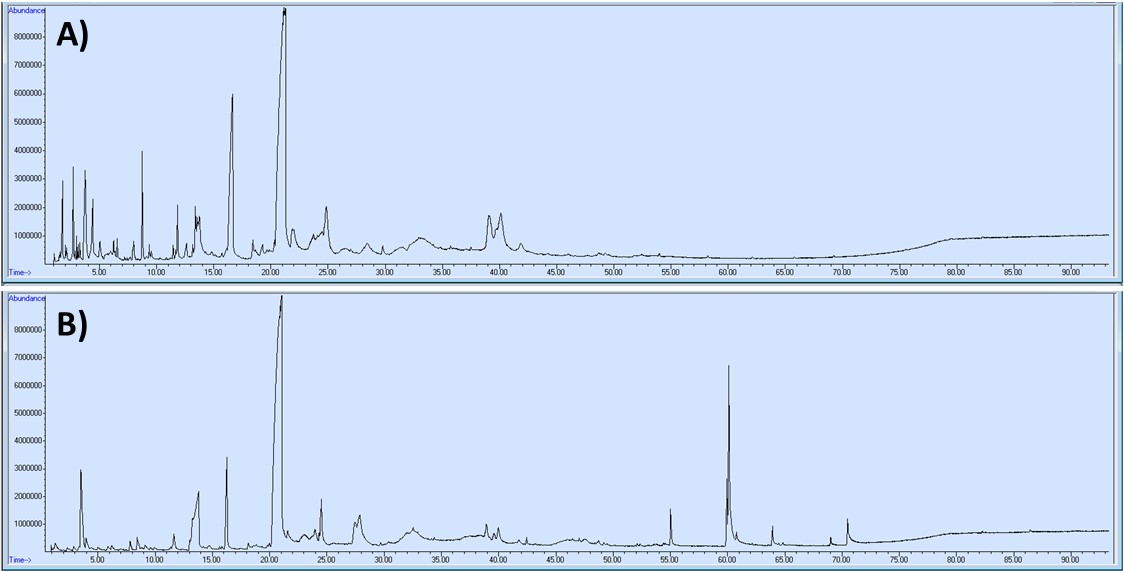


**Figure S1.** Representative ion chromatographs of a) healthy grape berries and b) grapes infected by *A. carbonarius* in day 8 after the appearance of conidia.
